# Transformed Shell Structures Determined by Regular Networks as a Complex Material for Roofing

**DOI:** 10.3390/ma14133582

**Published:** 2021-06-26

**Authors:** Jacek Abramczyk

**Affiliations:** Department of Architectural Design and Engineering Graphics, Rzeszow University of Technology, Al. Powstańców Warszawy 12, 35-959 Rzeszów, Poland; jacabram@prz.edu.pl

**Keywords:** regular systems of shells, parametric polyhedral networks, polygonal networks, division coefficients, corrugated shell roofs, complex substitute material, unconventional building free forms

## Abstract

The article presents a comprehensive extension of the proprietary basic method for shaping innovative systems of corrugated shell roof structures by means of a specific complex material that comprises regular transformable shell units limited by spatial quadrangles. The units are made up of nominally plane folded sheets transformed into shell shapes. The similar shell units are regularly and effectively arranged in the three-dimensional space in an orderly manner with a universal regular reference surface, polyhedral network, and polygonal network. The extended method leads to the increase in the variety of the designed complex shell roof forms and plane-walled elevation forms of buildings. For this purpose, the rules governing the creation of the continuous roof shell structures of many shells arranged in different unconventional visually attractive patterns and their discontinuous regular modifications are sought. To obtain several novel groups of similar unconventional parametric roof forms, single division coefficients and double division coefficients are used. The easy and intuitive modifications of the positions of the vertices belonging to the polygonal network on the side edges of the polyhedral network accomplished by means of a parametric algorithm allow one to adjust the geometry of the complete shell units to the geometric and material constraints related to the orthotropic properties of the transformed sheeting by means of these coefficients. The innovative approach to the shaping of the diverse unconventional roof structures requires the solving of many interdisciplinary problems in the field of mathematics, civil engineering, construction, morphology, architecture, mechanics, computer visualization, and programming.

## 1. Introduction

**Figure 1:**
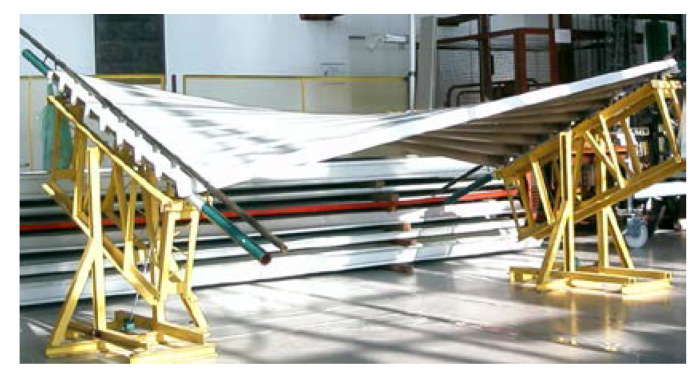
Experimental folded shell sheeting composed of eight folds belonging to two sheets supported by two straight skew directrices.

Small transverse compressive or tensile forces acting on a thin-walled nominally flat folded sheet can substantially alter the width of each fold of a sheet. The thin-walled, folded structure of the sheet allows each fold to change the width at its length in a diversified way. This property enables a strip of shell folds to take several forms of various ruled surfaces with a contraction appearing along the length of the shell fold [1] (Figure 1).

Symmetric elastic shape transformations of the thin-walled folded sheets ought to minimize their effort and allow one to obtain the highest possible degree of the shape changes generating the expected relatively big curvature of the transformed sheet while maintaining the ability to carry the characteristic roof load [2]. The rational forms of the transformed folded roof shells can be provided at the initial step of the process of modeling the buildings roofed with the transformed shells. For this purpose, various sectors of warped surfaces are created so that their contracting line runs halfway along the length of each of the transformed folds. It is not difficult to create shallow doubly-curved rectangular sectors of warped shells with negative Gaussian curvature due to the specific orthotropic geometric and material properties related to the shape transformations. These are mainly central sectors of hyperbolic paraboloids or quarters of these sectors.

**Figure 2:**
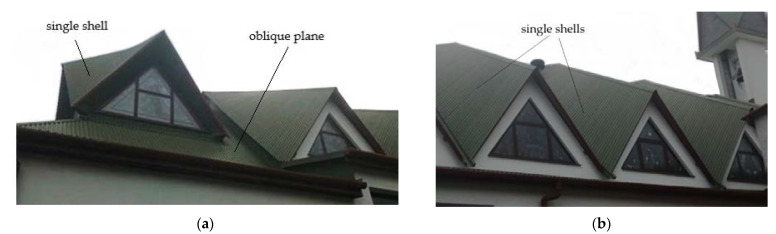
The external views of the same roof structure composed of several single shells arranged on an oblique plane: (**a**) the north side, (**b**) the south side.

In order to obtain a double-curved shell roof structure of medium or high positive Gaussian curvature, for example, an ellipsoid, it is necessary to form an unconventional shell roof structure composed of many similar shell units arranged on an auxiliary regular surface in the three-dimensional Euclidean space [1]. The so-called reference surface represents the general shell shape of a ribbed roof structure. The single shells of such a structure must be properly joined to each other along their edge lines (Figure 2).

In order to develop the rules governing the formation of several novel complex forms of the shell roofs that are impossible to obtain with the help of the single ruled shells due to the transformation constraints, methods based on spatial plane-walled reference networks are used [3,4]. One of such methods was developed and, next, expanded to utilize in elaborating the special rules defining different types of the complex shell roofs [5]. The considered roofs are derivatives of the basic roof shell structures described in the abovementioned articles.

**Figure 3:**
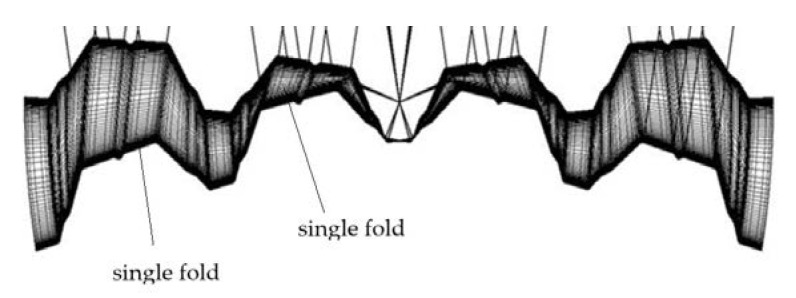
The accurate thin-walled computational mechanical model of a transformed sheet composed of four deformed shell folds.

For scientific research and engineering developments, several experimental tests and computer simulations (Figure 3) are carried out to analyze geometric and mechanical properties of thin-walled folded sheeting transformed into shell shapes [6,7].

## 2. Critical Analysis of the Present Knowledge

The research into the shape transformations of the nominally flat thin-walled corrugated steel sheets was initiated by Nilson in the 1970s. In the initial phase, the studies concerned the geometric and mechanical properties of complete shells transformed into the forms of central hyperbolic paraboloid sectors [8]. In order to increase the stiffness and critical loads of the transformed folded shells, two layers of folded sheets arranged in two orthogonal directions were used by the Winter team [9]. There were only created shallow double-layer shells characterized by a small transformation degree.

The degree can easily be increased by using single-layer sheeting at the expense of reducing the transverse stiffness of the transformed coating. Gioncu and Petcu developed a computer program for the calculation of the critical loads of the transformed shells [10]. The results of these tests point at the fact that the transformed single corrugated shells can be regarded as working in a membrane state.

**Figure 4:**
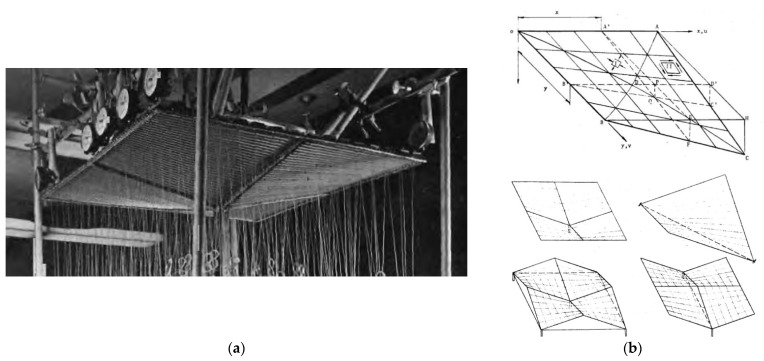
Hyperbolic paraboloid shells: (**a**) geometric models; (**b**) umbrella structures of four quarters.

The diversity, ridge, stiffness, and critical load of the transformed folds can significantly be increased by assembling several single shell units into one continuous ribbed shell roof structure (Figure 4a). The team led by Winter conducted comprehensive studies and published consistent results on the static-strength work of several single shells and shell structures composed of four quarters of the hyperbolic paraboloid central sectors arranged in different configurations (Figure 4b). Parker published the supplementary research results concerning the static-strength work of the complete coatings and [11].

The team led by Gergely presented a uniform description of the static-strength work of the single and complex transformed shells based on a relatively wide range of tests [12]. Simultaneously with the Gergely’s team, similar tests and analysis were conducted by Fisher et al. [13] in the field of static work and critical loads of the complete and complex hyperbolic paraboloid shells. The results of the tests and analysis of the work of the folded coatings were collected by Davis and Bryan to indicate the effective methods for shaping the shells [14].

The abovementioned researchers indicated the great theoretical possibilities of shaping various ruled forms made up of the transformed folded sheets with an open profile. Ultimately, on the basis of the tests and analysis carried out, they found that the encountered significant material and technological limitations drastically reduce the possibility of shaping the diversified shell forms of transformed sheeting to one basic type of the shallow hyperbolic paraboloids called hypars [12].

Therefore, it is reasonable to shape the ribbed structures composed of many single transformed shells by means of complex systems of complete ruled shells separated by sets of planes containing common or mutual displaced sections of the edge lines of these shells. Biswas and Iffland proposed a concept of such a system composed of many congruent transformed single shell units distributed over a sphere by means of a bundle of planes [15]. The transformed folded shells made up of aluminum or PVC “Selchim” corrugated plastic sheets were analyzed by Samyn [16]. The complete shells are shaped as revolved hyperboloids or right hyperbolic paraboloids limited by spatial quadrangles. Pottman proposed a comprehensive method of shaping the systems of planes separating subsequent smooth shell sectors in an arbitrary surface [17].

Reichhart developed a novel method for shaping the complete transformed thin-walled corrugated shells to increase their diversity, ridge, and transformation degree [18]. In accordance with Reichhart’s algorithm, the nominally flat single-layer sheeting is transformed into the position of the rigidly fixed directrices so that a freedom of the transverse width and height changes of all shell folds is assured. The previously mentioned methods did not provide such a freedom. Therefore, the effort of the transformed sheeting designed by means of these methods is significantly higher, and its critical load and ridge are significantly smaller compared to the shells formed by means of Reichhart’s method. Reichhart’s analysis carried out on the basis of the results of the tests allowed him to develop a method for calculating the shape, length, and mutual position of each shell directrix. The designed sheets do not have to be loaded with additional transverse forces to adjust their longitudinal edges or neutral axes to the positions of the selected rulings of an arbitrary ruled surface modeling the transformed roof shell.

On the basis of the results of the performed tests, Abramczyk noticed a significant role played by the contraction of each transformed shell sheeting in creating the effectively transformed thin-walled folded shells [1]. Abramczyk extended the Reichhart method and included the condition related to the central location of the contraction in the effectively transformed folded shells.

**Figure 5:**
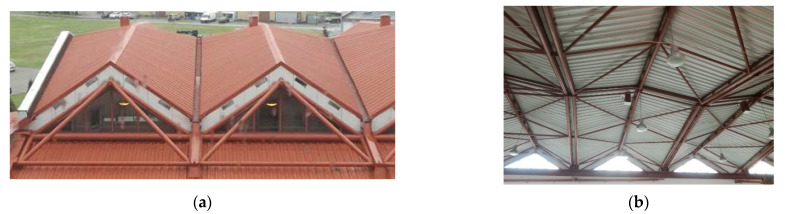
A shell structure of a few transformed shells roofing an experimental hall: (**a**) an outside view; (**b**) an inside view.

**Figure 6:**
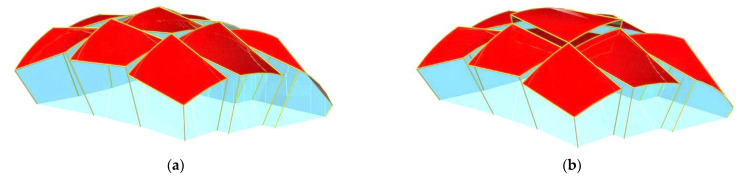
Two geometric models of building free forms roofed with complex corrugated shell sheeting structures with: (**a**) the rotated directrices of the central shell unit; (**b**) the translated directrices of the central shell unit.

Reichhart also developed a simple method of composing of a large number of identical complete transformed shells into a ribbed structure arranged on an oblique plane (Figure 2 and Figure 5) [19]. Abramczyk developed a much more complex method for regular arrangement of many complete shell units in the three-dimensional Euclidean space (Figure 6) based on the so-called reference surface [3]. The method results from the experimental studies and computer simulations of the transformed complete thin-walled folded shells [20]. It is possible to combine different shell units into one regular structure whose general form is similar to the arbitrary regular surface with almost free curvature using in constructions [21,22].

The properties of several thin-walled structures were detailed by Wei-Wen [23]. They can be used in shaping of the elastically transformed roof shells. Marin et al. [24] extended the classical theory of elasticity developed by Green and Lindsay in terms of the theory of thermo-elasticity for dipolar bodies. A novel method for a solution to a dynamical mixed problem was presented using a reciprocal theorem and not very restrictive conditions [25].

It is significant to investigate the space around the designed free-form building, its physical form and cultural patterns appearing in a whole spatial system. The relation between the formation of the urban space and the social experience of the human self was considered by Sharma [26]. The design syntax of urban greenways should also be taken into account. The mathematics-based graph studies of patterns and shapes, thermal based photography, and morphology to perform imagery-derived deductions on the design syntax were carried out by Hasgül [27].

Morphological shaping of buildings using many features specific to architectural, industrial, and structural design must be accomplished. Morphology is the study of the forms taking into account the relationships occurring between the function, structure, internal and external texture, static-strength work, and comfort conditions. Systematic morphology was defined by Eekhout as “the study of the system, rules and principles of form [that] has led to the interpretation of the study of the geometry of regular three-dimensional bodies or forms, usually known as polyhedra” and it plays a significant role in the design process [28]. The analogous universal systems of planes called polyhedral reference networks are utilized in this article.

## 3. The Aim

The main aim is to present the algorithm of an innovative extended method for shaping diversified unconventional systems of complex roof shell forms. The diversification is targeted at searching for unconventional basic and derivative configurations of the transformed shell roof structures. The research is related to the exploration of the essential dependencies dividing the shell structures into different groups of similar geometric properties and, next, the implementation of the obtained significant relations in the method’s algorithm. The complete transformed shell units characterized by the special orthotropic geometric and mechanical properties are used as a material for creating the shell roof structures. The units are made of flat thin-walled folded steel sheets transformed into similar shapes of ruled shells.

**Figure 7:**
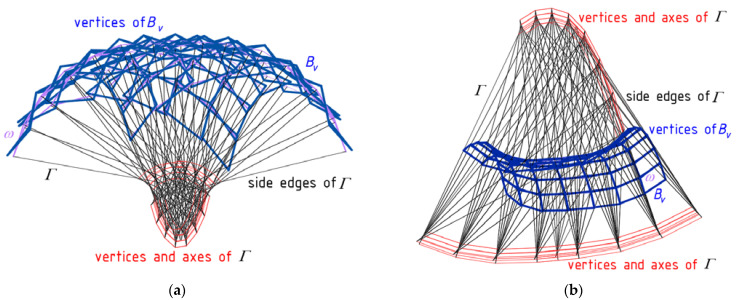
Two complex shell roof structures created on the basis of a polyhedral network *Γ*, a polygonal network *B_v_* and a reference surface *ω* characterized by: (**a**) the positive Gaussian curvature, (**b**) the negative Gaussian curvature.

The utilization of the method relies on creating two specific reference networks *Γ* and *B_v_* and adopting the set of division coefficients of the respective pairs of the vertices belonging to the first reference polyhedral network *Γ* by the vertices of the second reference polygonal network *B_v_* to determine several general forms of free-form buildings. The network *B_v_* defines the degree of the folding and discontinuity of the roof structure *Ω* (Figure 7a,b).

The formulas governing the mutual positions of the vertices of the subsequent meshes *B_vij_* (sectors *Ω_ij_*) in the required networks *B_v_* (structures *Ω*) must be invented. The expected types of the geometric patterns formed by *B_vij_* (*Ω_ij_*) on the network *B_v_* (structure *Ω*) have to result from the predicted relationships between the mutual positions of the vertices of the subsequent meshes *B_vij_* (sectors *Ω_ij_*). The proposed formulas must relate to the respective mutual displacements of the adjacent meshes *B_vij_* (sectors *Ω_ij_*) and their vertices along the side edges of the created polyhedral structure *Γ*, which allows one to achieve the appropriate type and form of the final derivative discontinuous roof structures *Ω*.

## 4. The Method’s Concept

The following concept of the research was adopted. The previously developed method was significantly extended and includes activities and objects that allow one to search for special rules relating to diversification of the visual patterns combined with many complete shells to achieve unconventional regular roof structures. The formulas governing the patterns result from the method of defining and arranging the single shell units in the three-dimensional space in an orderly and regular manner. For example, a reference surface and a set of division coefficients defining the location of the characteristic vertices of the designed structure can be utilized. The search for the relationships begins with the observation and description of the properties of the continuous ribbed shell roof structures.

**Figure 8:**
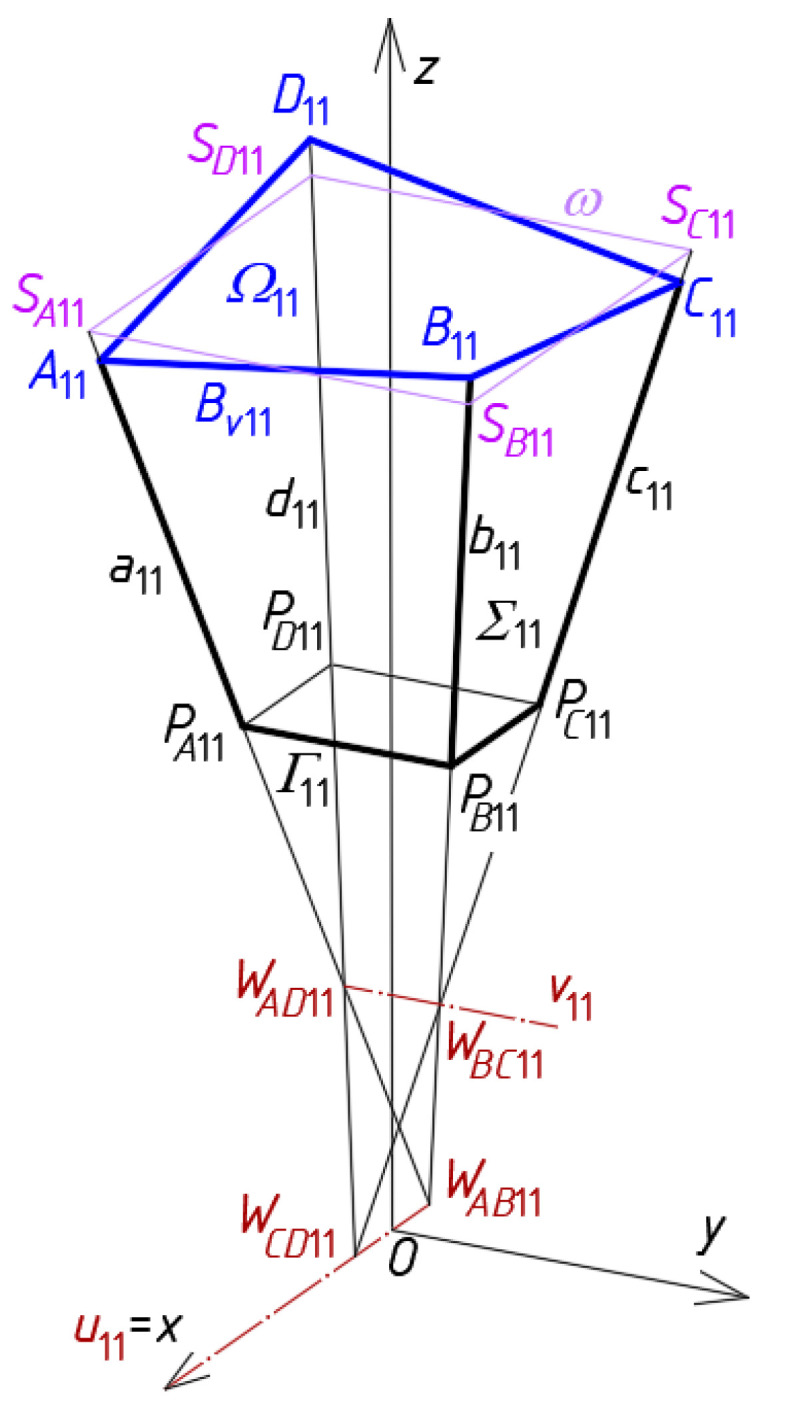
Properties of a complete central mesh *Γ*_11_ of a reference network *Γ*, a complete mesh *B_v_*_11_ of a quadrilateral eaves network *B_v_*, a plane-walled unit *Σ*_11_ of a free-form building structure Σ, and a central shell unit *Ω*_11_ of a discontinuous shell roof structure *Ω*.

For this purpose, a *z*-symmetric roof structure *Ω* consisting of two symmetrical and two antisymmetric parts is sought. The search starts with the determination of one symmetrical quarter of *Γ*. At the beginning, a single central shell *Ω*_11_ is created by means of a central tetrahedral mesh *Γ*_11_ and a central quadrilateral mesh *B_v_*_11_ (Figure 8). The subsequent meshes *Γ_ij_*, *B_vij_* and *Ω_ij_* of the nets *Γ*, *B_v_* and *Γ* are symmetrically arranged with respect to the *z*-axis-symmetric *Ω*_11_ in the orthogonal and diagonal directions.

The characteristic feature of the reference network *Γ* is that each single mesh *Γ_ij_* is a specific tetrahedron with four vertices *W_ABij_*, *W_CDij_*, *W_ADij_* and *W_BCij_*, two axes *u_ij_* and *v_ij_*, four side edges *a_ij_*, *b_ij_*, *c_ij_* and *d_ij_*, four triangular side walls (*W_ABij_W_CDij_W_BCij_*), (*W_ABij_W_CDij_W_ADCij_*), (*W_BCij_W_ADij_W_ABij_*) and (*W_BCij_W_ADij_W_CDij_*) contained in four planes defined by the above vertices. For the first mesh *i* = *j* = 1 (Figure 8).

In order to obtain the tetrahedron *Γ*_11_, the coordinates of its four vertices *W_AB_*_11_, *W_CD_*_11_, *W_AD_*_11_ and *W_BC_*_11_ must be defined based on a global coordinate system [*x,y,z*] [3,21]. The positions of the points *S_A_*_11_, *S_B_*_11_, *S_C_*_11_ and *S_D_*_11_ are defined with the following division coefficients d*_SA_*_11_ = (*W_AB_*_11_, *W_AD_*_11_)\*S_A_*_11_, d*_SB_*_11_ = (*W_AB_*_11_, *W_BC_*_11_)\*S_B_*_11_, d*_SC_*_11_ = (*W_CD_*_11_, *W_BC_*_11_)\*S_C_*_11_ and d*_SD_*_11_ = (*W_CD_*_11_, *W_AD_*_11_)\*S_D_*_11_ of the pairs (*W_AB_*_11_, *W_AD_*_11_), (*W_AB_*_11_, *W_BC_*_11_), (*W_CD_*_11_, *W_BC_*_11_) and (*W_CD_*_11_, *W_AD_*_11_), where

(1) $$(W_{AB11},W_{AD11})\backslash S_{A11}\;=m\left(\vec{W_{AB11}S_{A11}}\right)/m\left(\vec{W_{AB11}W_{AD11}}\right)\;(W_{AB11},W_{BC11})\backslash S_{B11}\;=m\left(\vec{W_{AB11}S_{B11}}\right)/m\left(\vec{W_{AB11}W_{BC11}}\right)\;(W_{CD11},W_{BC11})\backslash S_{C11}\;=m\left(\vec{W_{CD11}S_{C11}}\right)/m\left(\vec{W_{CD11}W_{BC11}}\right)\;(W_{CD11},W_{AD11})\backslash S_{D11}\;=m\left(\vec{W_{CD11}S_{D11}}\right)/m\left(\vec{W_{CD11}W_{AD11}}\right)$$

and $\vec{W_{AB11}W_{AD11}}$ is the vector starting with *W_AB_*_11_ and ending at *W_AD_*_11_, *m*($\vec{W_{AB11}W_{AD11}}$) is the measure of $\vec{W_{AB11}W_{AD11}}$, $\vec{W_{AB11}S_{A11}}$ is a vector with the starting point at *W_AB_*_11_ and the ending point at *S_A_*_11_, etc. The points *S_A_*_11_*, S_B_*_11_, *S_C_*_11_ and *S_D_*_11_ together with the analogous points assigned to the other meshes of *Γ* define the respective reference surface *ω*.

The locations of the vertices *A*_11_, *B*_11_, *C*_11_ and *D*_11_ of *Bv*_11_ (Figure 8) are defined by means of the vertices of *Γ*_11_ and the following proportions:

(2) $$d_{A11}\;=\;(W_{AB11},W_{AD11})\backslash S_{A11}\;=m\left(\vec{W_{AB11}S_{A11}}\right)/m\left(\vec{W_{AB11}W_{AD11}}\right)\;d_{B11}\;=\;(W_{AB11},W_{BC11})\backslash S_{B11}\;=m\left(\vec{W_{AB11}S_{B11}}\right)/m\left(\vec{W_{AB11}W_{BC11}}\right)\;d_{C11}\;=\;(W_{CD11},W_{BC11})\backslash S_{C11}\;=m\left(\vec{W_{CD11}S_{C11}}\right)/m\left(\vec{W_{CD11}W_{BC11}}\right)\;d_{D11}\;=\;(W_{CD11},W_{AD11})\backslash S_{D11}\;=m\left(\vec{W_{CD11}S_{D11}}\right)/m\left(\vec{W_{CD11}W_{AD11}}\right)$$

where $\vec{W_{AB11}A_{11}}$ is the vector with the starting point at *W_AB_*_11_ and the ending point at *A*_11_, etc. The points *A*_11_, *B*_11_, *C*_11_ and *D*_11_ determine the spatial quadrangle *B_v_*_11_ constituting the eaves of a single smooth shell segment *Ω*_11_ modeling a single shell of a complex roof structure.

The process of shaping of the reference network *Γ* consists in creating subsequent tetrahedrons characterized by common planes intersecting each other in axes and side edges. To define the networks *Γ* and *B_v_*, a set of the respective independent variables must be adopted and specific values have to be assigned to these variables. For all dependent variables, appropriate functions must be defined to determine the vertices of *Γ* and *B_v_*.

In order to obtain the tetrahedron *Γ*_12_ its four vertices *W_AB_*_12_, *W_CD_*_12_, *W_AD_*_12_ and *W_BC_*_12_ must be defined [3,29]. The vertex *W_AB_*_12_ = *W_CD_*_12_. Two next vertices can be calculated by means of two division coefficients as follows

(3) $$(W_{CD11},W_{BC11})\backslash W_{BC12}\;=\;m\left(\vec{W_{CD11}W_{BC12}}\right)/m\left(\vec{W_{CD11}W_{BC11}}\right)\;(W_{CD11},W_{AD11})\backslash W_{AD12}\;=\;m\left(\vec{W_{CD11}W_{AD12}}\right)/m\left(\vec{W_{CD11}W_{AD11}}\right)$$

where *m*($\vec{W_{CD11}W_{BC12}}$) is the measure of the vector $\vec{W_{CD11}W_{BC12}}$ with the starting point at *W_CD_*_11_ and the ending point at *W_BC_*_12_, and *m*($\vec{W_{CD11}W_{BC11}}$) is the measure of the vector $\vec{W_{CD11}W_{BC11}}$, etc.

**Figure 9:**
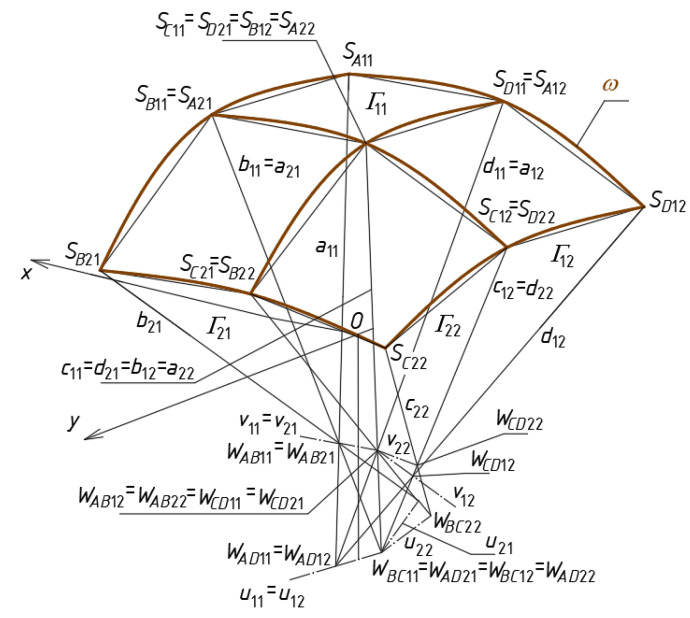
A reference regular surface *ω* based on the modified polyhedral network *Γ*.

The vertices of the other meshes *B_vij_* of *B_v_* should be defined in the same way as *B_v_*_11_ using formulas analogous to Equations (1)–(3). Each pair of two adjacent meshes *Γ_ij_* and *Γ_ij_*_+1_ or *Γ_ij_* and *Γ_i_*_+1*j*_ have one common side edge contained in a plane of *Γ*, for example, *Γ*_11_ and *Γ*_21_ has two common side edges *b*_11_ = *a*_21_ and *c*_11_ = *d*_21_, and three common vertices *W_BC_*_11_ = *W_AD_*_21_, *W_CD_*_11_ = *W_CD_*_21_ and *W_AB_*_11_ = *W_AB_*_22_ (Figure 9). Four adjacent meshes *Γ_ij_*, *Γ_ij_*_+1_, *Γ_i_*_+1*j*_ and *Γ_i_*_+1*j*+1_ have one common side edge *a_i_*_+1*j*+1_ = *b_i_*_+1*j*_ = *c_ij_* = *d_ij_*_+1_ of *Γ*, for example, *a*_22_ = *b*_12_ = *c*_11_ = *d*_21_ for *i* = *j* = 1.

**Figure 10:**
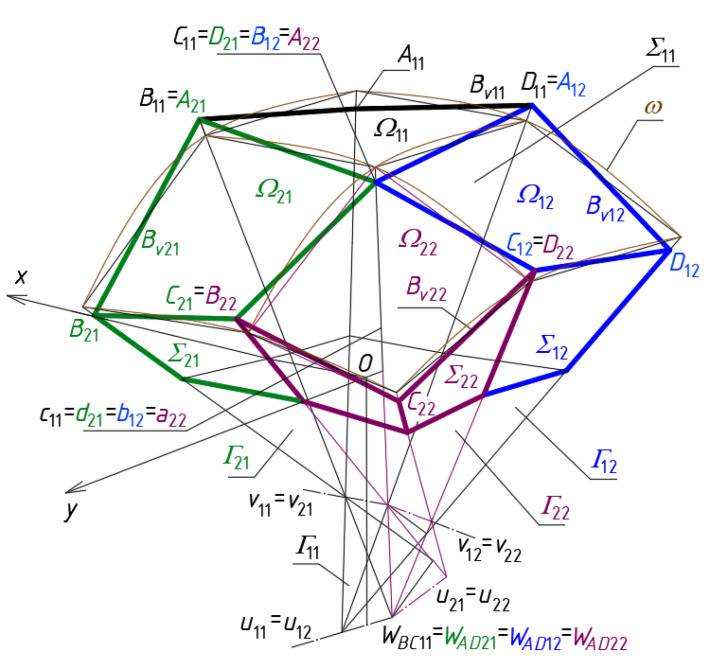
A quadrilateral eaves network *B_v_*, and a continuous ribbed shell roof structure *Ω*. defined on the basis of the polyhedral network *Γ*.

The subsequent quadrilateral meshes *B_vij_* of the targeted networks *B_v_* modeling the eaves of the examined shell roof structures are constructed based on the side edges of the auxiliary network *Γ*. Following to the method’s algorithm, the subsequent adjacent quadrilateral meshes of *B_v_* must have common vertices *A_ij_*, *B_ij_*, *C_ij_* or *D_ij_* determined on the edges *a_ij_*, *b_ij_*, *c_ij_* and *d_ij_* (Figure 9). The specific sum of all individual shells *Ω_ij_*, determined by means of *A_ij_*, *B_ij_*, *C_ij_* and *D_ij_,* constitutes a continuous ribbed roof structure *Ω* (Figure 10). Finally, a free-form Σ of many Σ*_ij_* is the simplified model of an entire complex building.

The complete tetrahedrons *Γ_ij_* can be regarded as an universal material for creating the spatial polyhedral networks modeling free-form building systems. Similarly, the single spatial quadrilateral meshes *B_vij_* can be accepted as a material for shaping the eaves systems *B_v_* of complex free-form roofs. In addition, the complete shell sectors *Ω_ij_* are used as a universal material for creating the free-form shell roof structures *Ω*. After all, the abovementioned systems create three subsequent layers producing one complex material used for shaping unconventional building free forms roofed with complex shell structures.

The article primarily presents the results of the research on the geometric properties of the eaves layer *B_v_* determining the form of the shell layer *Ω*. The observed properties are described with the help of the developed mathematical rules governing the systems of different patterns of the complete meshes *B_vij_* and sectors *Ω_ij_* arranged on *ω*. The results of these studies can be used in the design of several diversified unconventional free forms of buildings roofed with attractive and rational systems of many regular roof shells made up of transformed corrugated sheets.

**Figure 11:**
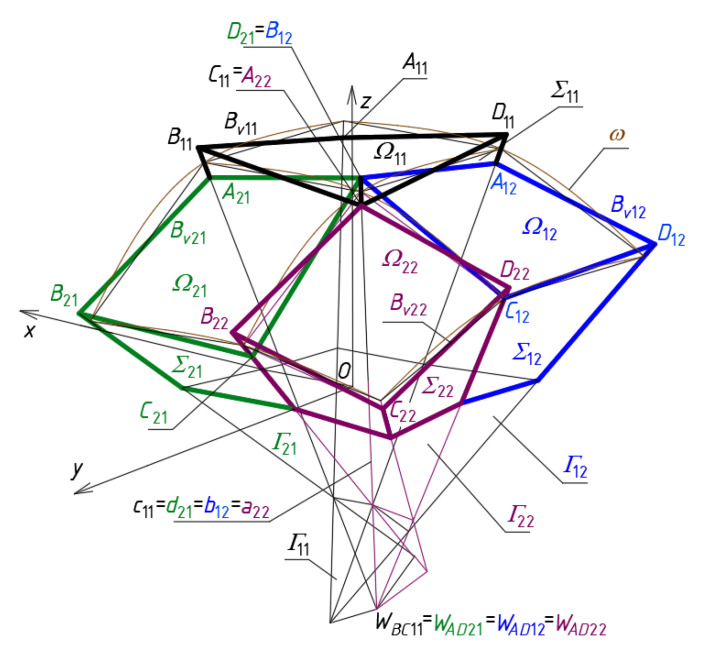
The first derivative configuration CP1 of a discontinuous shell roof structure *Ω*. defined on the basis of *Γ* and *B_v_*.

In order to carry out the research, the following algorithm was developed. It forbids two adjacent meshes of *B_v_* or segments of *Ω* to have common vertices and sides. Thus, a discontinuous net *B_v_* and a discontinuous structure *Ω* can be created as a result of a rotation of each pair of the respective eaves segments belonging to two adjacent shell units *Ω_ij_* and *Ω_i_*_+1*j*_ or *Ω_ij_*_+1_ (Figure 11). Four straight segments of each spatial quadrangle *B_vij_* are displaced in the respective planes of the network *Γ*. Thus, the basic continuous configuration CB and the modified discontinuous configuration CP1 of the structure *Ω* use the same network *Γ*.

However, such a separation of the positions of the vertices belonging to the selected pairs of two adjacent meshes of *B_v_* (*Ω*) requires appropriate changes in the values of the division coefficients assigned to the respective vertices of *Γ* of each *B_vij_*. The goal of the research is therefore to develop a method of modifying the values of the coefficients so that different groups of discontinuous shell roof structures can be achieved. This activity requires defining some uniform conditions and adopting mathematical formulas describing the modified net *B_v_* and structure *Ω*.

**Figure 12:**
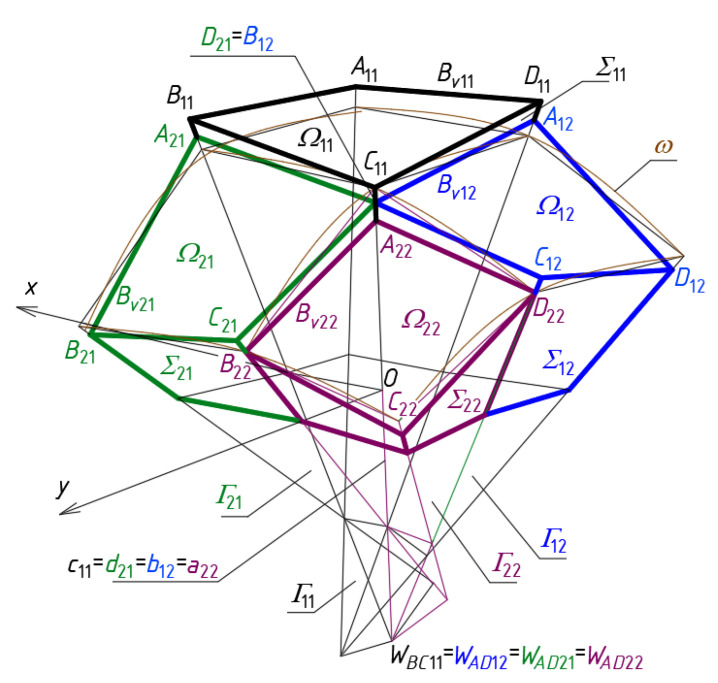
The second derivative configuration CP2 of a discontinuous shell roof structure *Ω*. defined on the basis of *Γ* and *B_v_*.

The designed discontinuous nets *B_v_* and structures *Ω* can also be created as a result of translations of two respective eaves segments belonging to two adjacent shell units *Ω_ij_* and *Ω_i_*_+1*j*_ or *Ω_ij_*_+1_ (Figure 12). Four straight segments of each quadrangle *B_vij_* can be displaced in the respective planes of *Γ*.

Finally, the following algorithm based on four general steps should be adopted. The main characteristic of the algorithm is the usage of the division coefficients of the vertices of the reference polyhedral network *Γ* by the vertices of a reference polygonal network *B_v_* (Figure 7) and the selected points of a reference surface *ω*. At the first step of the algorithm, the division coefficients are used to obtain the reference polyhedral network *Γ* and the repeatability of its tetrahedral meshes *Γ_ij_*, especially in the orthogonal directions. At the second step of the algorithm, the division coefficients are used to determine several points defining the reference surface *ω*. The expected curvature and general form of the shell roof structure are designated in an easy and intuitive way by means of the relations defined by means of the arbitrary division coefficients.

The division coefficients are also used at the third step of the method’s algorithm to determine the vertices of the reference polygonal network *B_v_*. The vertices are located on the side edges of the polyhedral reference network *Γ* and defined with respect to the reference surface *ω*. The obtained vertices define the polygonal network *B_v_* composed of quadrilateral spatial meshes *B_vij_*. Four segments of *B_vij_* determine one complete shell unit *Ω_ij_* and constitute its eaves line. At this step, a basic configuration CB of the sought-after shell roof structure *Ω* is created. The configuration is composed of many individual shells *Ω_ij_* so that the roof structure *Ω* is continuous (Figure 4b, Figure 5a,b and Figure 10). Each pair of two adjacent complete shells of the structure shares one edge.

The network *Γ* and the network *B_v_* determine the curvature and folding of the roof structure *Ω*. The curvature of *Ω* can be determined using the properties of *ω*. The folding of *Ω* results from the ribs existing between the adjacent complete smooth shell units *Ω_ij_*.

The fourth step of the method’s algorithm allows one to create several derivative configurations of the examined continuous roof structure. The derivative configurations are most often characterized by a mutual translation or rotation of the edge lines of the adjacent shells (Figure 6a,b, Figure 11 and Figure 12) in the planes of *Γ*. The discussion of the activities and their effects provided for in this step of the algorithm is the essential part of the research presented in this article.

The accuracy of the algorithm is comparable with the accuracy of the adopted data, and it is equal to 1 mm. This results from the 0.5 mm accuracy of the performed experimental tests and the 1 mm accuracy of the calculations of the ruling’s position of each complete shell *Ω_ij_* of *Ω*.

## 5. Results

**Figure 13:**
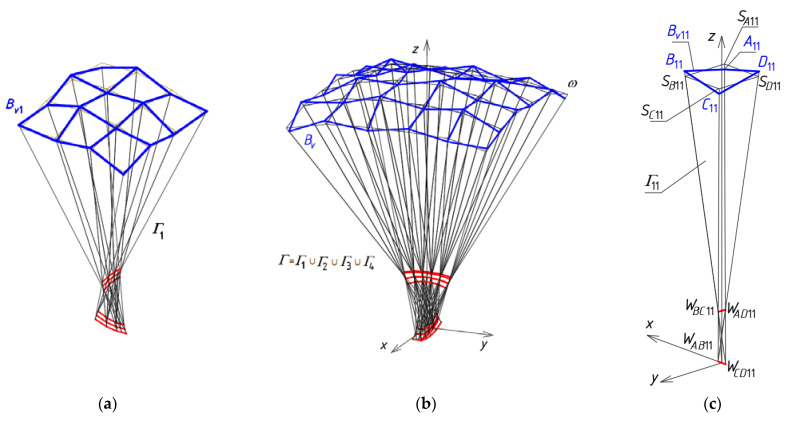
The polyhedral and quadrilateral nets for a continuous roof structure: (**a**) the one-fourth *Γ*_1_ of *Γ* and the one-fourth *B_v_*_1_ of *B_v_*; (**b**) the entire *Γ* and *B_v_* networks; (**c**) the central meshes *Γ*_11_ and *B_v_*_11_.

The search for the rules governing the systems of the diversified patterns of the complete transformed shells on the roof structures is presented in three examples of networks *B_v_* using the investigated algorithm. At the first step of the algorithm, the axes *u_ij_* and *v_ij_*, side edges *a_ij_*, *b_ij_*, *c_ij_* and *d_ij_*, and the entire *Γ* are determined on the basis of the adopted vertices *W_ABij_*, *W_CDij_*, *W_ADij_* and *W_BCij_* [3,21]. This step is divided into two sub-steps requiring the designer to create: (1) the mesh *Γ*_11_, meshes *Γ_i_*_1_ and *Γ*_1*j*_ (for *i* > 1 and *j* > 1) located orthogonally in relation to *Γ*_11_, (2) meshes *Γ_ij_* located diagonally relative to *Γ*_11_, *Γ_i_*_1_ or *Γ*_1*j*_. The sub-steps differ from each other in terms of the initial data and the actions necessary to build the subsequent single meshes *Γ_ij_*. The values of the coordinates of the vertices belonging to the *Γ*_1_s (Figure 13a) are presented in Table A1 in Appendix A.

At the second step of the algorithm, all points defining a reference surface *ω* are determined. In this way, the size of *Γ* and the curvature of *ω* are defined. For this purpose, the values of the selected division coefficients are adopted and the coordinates of the points *S_Aij_*, *S_Bij_*, *S_Cij_* and *S_Dij_* defining the reference surface *ω* are calculated using Equation (1) and the initial data are published in Table A2 in Appendix A. The coordinates of these points are published in Table A3 in Appendix A.

At the third step, vertices of all quadrilateral meshes of the designed *B_v_* for the basic configuration CB are defined on the side edges of *Γ* relative to *ω* (Figure 13b). At this step, the complete shell segments *Ω_ij_* of an entire roof structure *Ω* constituting the basic configuration CB are defined on the basis of *B_vij_*. The adopted values of the division coefficients d*_Aij_*, d*_Bij_*, d*_Cij_* and d*_Dij_* used for achieving the *B_v_* vertices are presented in Table A4 in Appendix A. The coordinates of the *Bv* and *Ω* vertices calculated with the help of Equation (2) are presented in Table A5 in Appendix A.

The values of four division coefficients d*_SijAij_* = (*W_ABij_*,*W_ADij_*)\(*S_Aij_*,*A_ij_*), d*_SijBij_* = (*W_ABij_*,*W_BCij_*)\(*S_Bij_*,*B_ij_*), d*_SijCij_* = (*W_CDij_*,*W_BCij_*)\(*S_Cij_*,*C_ij_*) and d*_SijDij_* = (*W_CDij_*,*W_ADij_*)\(*S_Dij_*,*D_ij_*) are calculated to estimate the folding of *Ω* (*B_v_*) related to the diversification of the locations of the vertices of *B_v_* relative to *ω*. The coefficients are used with the positive or negative sign depending on whether the points *A_ij_*, *B_ij_*, *C_ij_* and *D_ij_* lie above or below *ω* defined by means of the respective quadrangle *S_Aij_S_Bij_S_Cij_S_Dij_*. The calculated coordinates of the vertices *A_ij_*, *B_ij_*, *C_ij_* and *D_ij_* are presented in Table A6 in Appendix A.

The ratios d*_S_*_11*A*11_ = (*W_AB_*_11_,*W_AD_*_11_)\(*S_A_*_11_,*A*_11_), d*_S_*_11*B*11_ = (*W_AB_*_11_,*W_BC_*_11_)\(*S_B_*_11_,*B*_11_), d*_S_*_11*C*11_ = (*W_CD_*_11_,*W_BC_*_11_)\(*S_C_*_11_,*C*_11_) and d*_S_*_11*D*11_ = (*W_CD_*_11_, *W_AD_*_11_)\(*S_D_*_11_,*D*_11_) are defined for *B_v_*_11_ as follows:

(4) $$d_{S11A11}\;=\;m\left(\vec{S_{A11}A_{11}}\right)/m\left(\vec{W_{AB11}W_{AD11}}\right)=d_{A11}-d_{SA11}\;d_{S11B11}\;=\;m\left(\vec{S_{B11}B_{11}}\right)/m\left(\vec{W_{AB11}W_{BC11}}\right)=d_{B11}-d_{SB11}\;d_{S11C11}\;=\;m\left(\vec{S_{C11}C_{11}}\right)/m\left(\vec{W_{CD11}W_{BC11}}\right)=d_{C11}-d_{SC11}\;d_{S11D11}\;=\;m\left(\vec{S_{D11}D_{11}}\right)/m\left(\vec{W_{CD11}W_{AD11}}\right)=d_{D11}-d_{SD11}.$$

The above division coefficients allow one to observe the absolute differences in the mutual positions of the points *A*_11_, *B*_11_, *C*_11_ and *D*_11_ (Figure 13c). The obtained values can easily be converted into the distances of these points from *ω*. The coordinates of the *B_v_* vertices calculated with the help of the equations analogous to Equation (4) are presented in Table A5 in Appendix A.

The coefficients d*_SijAij_*, d*_SijBij_*, d*_SijCij_* and d*_SijDij_* do not give the precise information about the folding of the network *B_v_* (structure *Ω*) because they express the proportions in relation to the distance of two adjacent vertices of *Γ*. Therefore, the proportions signifying the position of the points *A_ij_*, *B_ij_*, *C_ij_* and *D_ij_* in relation to the position of the points *S_Aij_*, *S_Bij_*, *S_Cij_*, *S_Dij_* and the position of the vertices of *Γ* must be preferred to present the folding more precisely.

Thus, the following double division coefficients play an important role in describing the geometrical properties of the roof structures:

d_*ASij*_ = (*W_AB__ij_*,*W_AD__ij_*)\(*A_ij_/S_A__ij_*) = d_*Aij*_/d_*SAij*_ d_*BSij*_ = (*W_AB__ij_*,*W_BC__ij_*)\(*B_ij_*/*S_B__ij_*) = d_*Bij*_/d_*SBij*_ d_*CSij*_ = (*W_CD__ij_*,*W_BC__ij_*)\(*C_ij_/S_C__ij_*) = d_*Cij*_/d_*SCij*_ d_*DSij*_ = (*W_CD__ij_*,*W_AD__ij_*)\(*D_ij_*/*S_D__ij_*) = d_*Dij*_/d_*SDij*_. (5)

If the values calculated by means of Equation (5) are greater than one, then the respective points lie above the surface *ω*. If the values are less than one, then the points lie below *ω*.

The following double division coefficients are a much more convenient and intuitive variable describing the folding of a shell roof structure:

d_*A*\*Sij*_ = (d_*Aij*_ − d_*SAij*_)/d_*SAij*_ d_*B*\*Sij*_ = (d_*Bij*_ − d_*SBij*_)/d_*SBij*_ d_*C*\*Sij*_ = (d_*Cij*_ − d_*SCij*_)/d_*SCij*_ d_*D*\*Sij*_ = (d_*Dij*_ − d_*SDij*_)/d_*SDij*_. (6)

The new division coefficients show the relative proportions of the folding of *B_v_* (*Ω*) in relation to the positions of *ω* and the respective vertices of *Γ*. The values of these coefficients were calculated by means of Equation (6) for the selected meshes of *B_v_*. They are presented in Table A7 in Appendix A.

The polyhedral reference networks *Γ*, reference surfaces *ω*, polygonal networks *B_v_* and shell roof structures *Ω* are the main geometric objects created by means of the investigated method in the process for shaping building free forms roofed with complex transformed corrugated shell structures. The network *B_v_* is the most important because the edge lines of all its complete meshes *B_vij_* determine all individual shell segments *Ω_ij_* of the resultant roof structure *Ω*. The *B_v_* sides are the eaves of *Ω_ij_*. To create a network *B_v_* characterized by the expected properties, the method introduces the auxiliary reference polyhedral network *Γ* assisting and facilitating the determination of the positions of the vertices *A_ij_*, *B_ij_*, *C_ij_* and *D_ij_* of *B_v_*.

The main property of each reference network *Γ* is that each pair of its adjacent side edges must intersect. The intersecting points of the respective pairs of two adjacent side edges denoted as *a_ij_*, *b_ij_*, *c_ij_* or *d_ij_* (Figure 8 and Figure 9) are called vertices *W_ABij_*, *W_CDij_*, *W_BCij_* and *W_ADij_* of *Γ*. The edges define all planes of *Γ*. It is very important that two adjacent meshes of *B_v_* have one common segment of their edge lines (for continuous structures *Ω*) or two different segments (for discontinuous structures *Ω*) contained in the same plane of *Γ*, so the segments are coplanar. This property significantly simplifies the processes for shaping of the regular basic continuous configurations of *Ω* and their derivative configurations.

The research on searching for the rules governing the locations of the *B_v_* vertices and the patterns of the complete *Ω_ij_* shells starts with the analysis of the properties of the basic configuration CB of the shell structures shown in Figure 10 and Figure 13. The configuration CB allows one to create a so-called continuous roof structure *Ω*. This configuration is characterized by the fact that each four adjacent quadrangles *B_vij_*, *B_vi_*_+1*j*_, *B_vij_*_+1_ and *B_vi_*_+1*j*+1_ have one common vertex *C_ij_* = *B_ij_*_+1_ = *D_i_*_+1*j*_ = *A_i_*_+1*j*+1_. The process of the creation of the new polygonal networks *B_v_* derivative of the basic configuration CB is relatively simple because the sides of *B_vij_* are displaced in the abovementioned planes of *Γ*. Similarly, the *B_v_* vertices belong to the side edges of *Γ* during these displacements.

**Figure 14:**
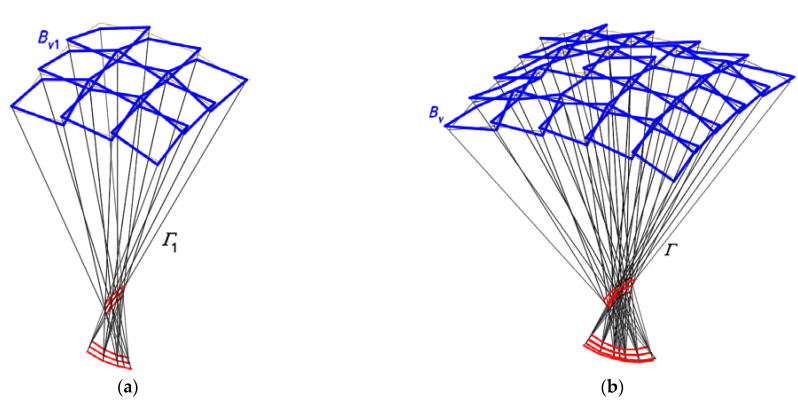
The polyhedral and quadrilateral nets for a discontinuous roof structure: (**a**) one-fourth of *Γ*_1_ and *B_v_*_1_; (**b**) entire *Γ* and *B_v_*.

**Figure 15:**
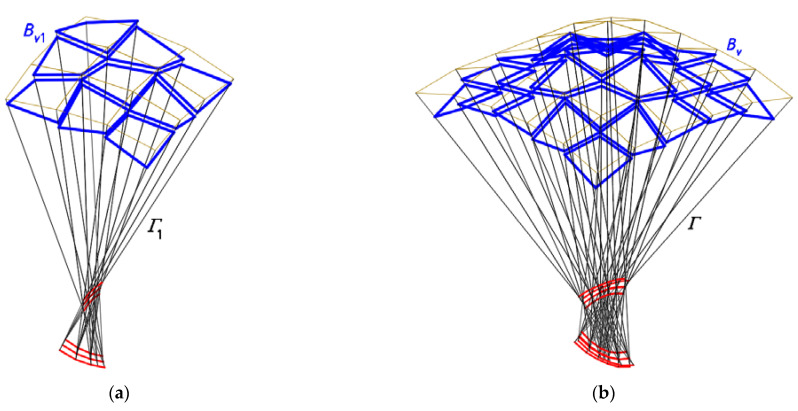
The results of the further steps of the method’s algorithm used for creating *Γ*: (**a**) *Γ*_21_; (**b**) *Γ*_22_.

The goal of this article is to focus on the next step of the method’s algorithm related to some modifications of the basic nets *B_v_*. In particular, the activities forcing a diversification of the positions of the vertices of four adjacent *B_v_* meshes corresponding to each other are analyzed. The change of the positions of the *B_v_* vertices consists in varying their positions on the side edges of *Γ* in relation to *ω*. These modifications lead to diversified and original patterns of *B_vij_* and *Ω_ij_* on *B_v_* and *Ω* (Figure 14 and Figure 15).

The first derivative configuration CP1 defines a discontinuous shell structure *Ω* and a discontinuous polygonal network *B_v_* characterized by flat areas of discontinuity between the subsequent shell sectors *Ω_ij_* of *Ω* (Figure 11 and Figure 14a,b). The discontinuous areas occurring between each pair of two adjacent meshes *B_vij_* and *B_vi_*_+1*j*_ (*Ω_ij_* and *Ω_i_*_+1*j*_) or *B_vij_* and *B_vij_*_+1_ (*Ω_ij_* and *Ω_ij_*_+1_) are the combinations of various triangles contained in the *Γ* planes. For this configuration, all points *A_ij_* and *C_ij_* are located below *ω* and all points *B_ij_* and *D_ij_* are located above *ω* at the distances resulting from the respective values of the division coefficients d*_Aij_*, d*_Bij_*, d*_Cij_* and d*_Dij_*. The configuration CP1 is characterized by tetrads of the adjacent meshes *B_vij_*, *B_vi_*_+1*j*_, *B_vij_*_+1_ and *B_vi_*_+1*j*+1_ with two pairs of common vertices: *C_ij_* = *A_i_*_+1*j*+1_ and *B_ij_*_+1_ = *D_i_*_+1*j*_ located independently on the same side edge *c_ij_* = *b_ij_*_+1_ = *d_i_*_+1*j*_ = *a_i_*_+1*j*+1_ of *Γ*.

The first spatial quadrangle *B_v_*_11_ is defined so that the points *A*_11_ and *C*_11_ lie on the side edges *a*_11_ and *c*_11_ below *ω* and the points *B*_11_ and *D*_11_ above *ω* at the distances used for the basic configuration CB and resulting from the adopted values of the following division coefficients: d*_S_*_11*A*11_ = d*_S_*_11*C*11_ = −0.1 and d*_S_*_11*B*11_ = d*_S_*_11*D*11_ = 0.1. The positions of two next meshes *B_v_*_12_ and *B_v_*_21_ are obtained by moving the points *A*_12_, *B*_12_, *C*_12_, *D*_12_, *A*_21_, *B*_21_, *C*_21_ and *D*_21_ of the configuration CB along the respective side edges of the *Γ* network to their new positions *A*_12_, *B*_12_, *C*_12_, *D*_12_, *A*_21_, *B*_21_, *C*_21_ and *D*_21_ of the new configuration CP1 at the distances resulting from the values of the respective division coefficients adopted for *B_v_* and *Γ*. The values of the coordinates of the vertices belonging to the quarter *Γ*_1_ of the *z*-axis-symmetric network *Γ* calculated for CP1 are presented in Table A8 in Appendix A.

The second configuration CP2 derivative of CB is characterized by many flat quadrilateral areas of discontinuity between adjacent meshes *B_vij_*, *B_vi_*_+1*j*_, *B_vij_*_+1_ and *B_vi_*_+1*j*+1_ (Figure 12 and Figure 15). The locations of its vertices *A_ij_*, *B_ij_*, *C_ij_* and *D_ij_* defining the quadrangular areas of the *Ω* discontinuity can be found as follows.

The first quadrangle *B_v_*_11_ is the same as for the basic configuration CB and derivative configuration CP1. The positions of two following quadrangles *B_v_*_12_ and *B_v_*_21_ are obtained by moving the points *A*_12_, *B*_12_, *C*_12_, *D*_12_, *A*_21_, *B*_21_, *C*_21_ and *D*_21_ of the configuration CB along the respective side edges of the *Γ* network into their new positions *A*_12_, *B*_12_, *C*_12_, *D*_12_, *A*_21_, *B*_21_, *C*_21_ and *D*_21_ of the new configuration CP2 at the distances resulting from the values of the division coefficients d*_S_*_12*A*12_, d*_S_*_12*B*12_, d*_S_*_12*C*12_ and d*_S_*_12*D*12_, etc. The values of the coordinates of the vertices belonging to the quarter *Γ*_1_ are presented in Table A9 in Appendix A.

The positions of the other vertices belonging to the subsequent new quadrangles *B_v_*_13_, *B_v_*_22_ *B_v_*_23_, *B_v_*_32_ and *B_v_*_31_ of CP2 are obtained as a result of moving the points *A*_13_, *B*_13_, *C*_13_, *D*_13_, *A*_22_, *B*_22_, *C*_22_, *D*_22_, *A*_23_, *B*_23_, *C*_23_, *D*_23_, *A*_31_, *B*_31_, *C*_31_, *D*_31_, *A*_32_, *B*_32_, *C*_32_ and *D*_32_ of CB along the respective side edges of *Γ* into their new positions of CP2 resulting from the respective values of the division coefficients d*_SijAij_*, d*_SijBij_*, d*_SijCij_* and d*_SijDij_* for *i* = 1 and *j* = 3 or *i* = 3 and *j* = 1. In particular, the coefficients can be equal to twice or three times the value of the respective coefficient d*_S_*_11*A*11_ or d*_S_*_11*B*11_ or d*_S_*_11*C*11_ or d*_S_*_11*D*11_.

## 6. Discussion

All vertices *A_ij_*, *B_ij_*, *C_ij_* and *D_ij_* of the basic configuration CB are divided into two groups. The first group includes the vertices lying on one side of a reference surface *ω*, for example, above *ω*. The second group is composed of the other *B_v_* vertices lying on the opposite side of *ω*, that is, under *ω*.

The positions of the sought-after vertices *A_ij_*, *B_ij_*, *C_ij_* and *D_ij_* lying on the side edges *a_ij_*, *b_ij_*, *c_ij_* and *d_ij_* of *Γ* result from the adopted or calculated values of the division coefficients d*_SAij_*, d*_SBij_*, d*_SCij_* and d*_SDij_*. If we know the values of the above coefficients, the values the division coefficients d*_Aij_*, d*_Bij_*, d*_Cij_* and d*_Dij_*, required to calculate the positions of *A_ij_*, *B_ij_*, *C_ij_* and *D_ij_*, can be calculated as follows:

d_*Aij*_ = d_*SAij*_ + d_*SijAij*_ d_*Bij*_ = d_*SBij*_ + d_*SijBij*_ d_*Cij*_ = d_*SCij*_ + d_*SijCij*_ d_*Dij*_ = d_*SDij*_ + d_*SijDij*_. (7)

The values of the division coefficients d*_Aij_*, d*_Bij_*, d*_Cij_* and d*_Dij_* corresponding to the vertices of CB can be calculated with Equation (7) and the values are published in Table A1 and Table A5 in Appendix A.

In the case of the investigated basic configuration CB (Figure 10 and Figure 13), the first subset is composed of the points *A_ij_* and *C_ij_* located under *ω*, whereas the second subset is composed of the other points *A_ij_* and *C_ij_* positioned above *ω*. Similarly, some of the points *B_ij_* and *D_ij_* are located above *ω* and others lie under *ω*.

To divide the points *A_ij_* and *C_ij_* into two complementary subsets, the following formulas were developed. The points *A_ij_* and *C_ij_* are located under *ω* if the subscripts *i* and *j* meet the following conditions:

*i = j* + 2 ∙ *k_C_* for *i* > *j* (8)

or

*j* = *i* + 2 ∙ *k_C_* for *i* < *j* (9)

or

*i* = *j* (10)

where *k_C_* is the integer constant corresponding to the examined mesh *B_vij_*. If Equations (8)–(10) related to the values of *i* and *j* of the respective mesh *Ω_ij_* are fulfilled, the points *B_ij_* and *D_ij_* are located above *ω*. For example, if *k_C_* = 0 and *j* = 1, then *i* = 3 and the points *A*_31_ and *C*_31_ lie below *ω* and the points *B*_31_ and *D*_31_ lie above *ω*. The same result is achieved when adopting *k_c_* = 0 and *i* = 1. For this case, *j* = 3 and the points *A*_13_ and *C*_13_ lie below the surface *ω* and the points *B*_13_ and *D*_13_ lie above *ω*. If we use Equation (10) and *i* = *j* = 3, then we obtain the points *A*_33_ and *C*_33_ lying below *ω*.

The points *A_ij_* and *C_ij_* take the positions above *ω* and the points *B_ij_* and *D_ij_* lie under *ω* if the following conditions are met:

*i =* 1 + *j* + 2 ∙ *k_C_* for *i* > *j* (11)

or

*j* = 1 + *i* + 2 ∙ *k_C_* for *i* < *j*. (12)

For example, if *k_C_* = 0 and *j* = 1, then it follows from Equation (13) that *i* = 2 and the points *A*_21_ and *C*_21_ lie above *ω*, and the points *B*_21_ and *D*_21_ lie below *ω*. The same result is achieved when using Equation (12) and adopting *k_C_* = 0 and *i* = 1. In this case, *j* = 2 and the points *A*_12_ and *C*_12_ lie above *ω*, and the points *B*_12_ and *D*_12_ lie below *ω*.

The coefficients express the proportions between the diversity of the positions of the *B_v_* vertices in relation to *ω*, and the diversity of the locations of the same vertices in relation to the *Γ* vertices. They allow one to describe and parameterize the form of not only the roof structure, but also the form of the entire building, including the attractiveness and proportions between its basic dimensions. In this case, the double division coefficients must relate to the base level of the designed building. The above issues go beyond the scope of the article.

Thus, each of four vertices of *B_vij_* of each basic configuration CB is shared with three adjacent meshes. In the search for several discontinuous configurations derivative of the base configuration CB, it is advisable to analyze the number of the adjacent *B_v_* meshes possessing a common vertex. Four, three, two or only one vertex may be shared by the mesh with four adjacent meshes. The order of the common vertices of the adjacent meshes also affects the diversification of the derivative configurations.

The first derivative configuration CP1 (Figure 14) examined in the previous section is characterized by the fact that two *B_vij_* and *B_vi_*_+1*j*+1_ subsequent meshes of *B_v_* arranged in the diagonal directions have one common vertex. The meshes arranged in the orthogonal directions do not have such a common vertex, and the respective sides of two adjacent meshes are mutually rotated in the planes of *Γ*.

The conditions determining the positions of the vertices belonging to each tetrad of the adjacent *B_v_* meshes of the configuration CP2 are adopted so that a complete separation of the common vertices of the basic configuration CB is achieved. Thus, no pair of the adjacent meshes of CP2 has common vertices, and one side of each pair of two adjacent *B_v_* meshes is displaced in the respective plane of *Γ*. As a result, a discontinuous roof structure consisting of many single transformed shells *Ω_ij_* limited by the mutually rotated eaves *B_vij_* is built (Figure 12 and Figure 15). The displacement is accomplished by simultaneously moving two ends of the above side along the respective side edges of *Γ* either in the directions of the respective *Γ* vertices or in the opposite directions. This action causes the additional increments of the division coefficients d*_SijAij_*, d*_SijBij_*, d*_SijCij_* and d*_SijDij_* resulting from the movement to be of the same sign.

For the special case when the values of these increments are identical, it is necessary to consider the number of the modifications accomplished for the subsequent pairs of two adjacent *B_v_* meshes when transmitting from *B_v_*_11_ to the examined *B_vij_*. The transition from *B_v_*_11_ to *B_v_*_23_ requires three skips between *B_v_*_11_ and *B_v_*_12_, *B_v_*_12_ and *B_v_*_13_, and *B_v_*_13_ and *B_v_*_23_. Thus, in order to build a mesh *B_vij_*, the number *i* + *j* − 2 of the skips is required. If the values of the increments assigned to the division coefficients d*_SAij_*, d*_SBij_*, d*_SCij_* and d*_SDij_* of CP2 are equal to the same constant dd*_ij_*, then the increments dd*_Aij_,* dd*_Bij_,* dd*_Cij_* and dd*_Dij_* used for the vertices *A_ij_*, *B_ij_*, *C_ij_* and *D_ij_* (in relation to the base configuration CB) can be calculated from the formula

dd_*Aij*_ = dd_*Bij*_ = dd_*Cij*_ = dd_*Dij*_ = (*i* + *j* − 2) ∙ dd_*ij*_ (13)

where dd*_ij_* is the arbitrary constant related to the abovementioned skips.

It should be noted that two selected vertices of each pair of the adjacent meshes of *B_v_* arranged in the diagonal directions (Figure 15), for example, *B_v_*_21_ and *B_v_*_12_, have one common vertex. If we change the values of the coefficients d*_SijAij_*, d*_SijBij_*, d*_SijCij_* and d*_SijDij_* of all *B_vij_* to create the configuration CP2 (in relation to the basic configuration CB) using Equation (13), then we should obtain the identical location of the vertices of the adjacent diagonal meshes *B_vij_* and *B_vi_*_-1*j*+1_ only for the very specific mutual position of all vertices *W_ABij_*, *W_CDij_*, *W_BCij_* and *W_ADij_* of *Γ*. In fact, it will not be possible to achieve the abovementioned property of CP2 if we change the positions of these vertices of *Γ*. This issue goes beyond the scope of the article.

## 7. Conclusions

The method for creating discontinuous configurations derivative of the specific continuous basic configurations of the transformed shell roof structures defined by means of the systems of planes is proposed due to the necessity to assemble many complete transformed shells into one roof structure *Ω* resulting from the material limitations of the transformed corrugated sheeting. The method uses two specific reference networks *Γ* and *B_v_* and the proposed set of the division coefficients of the respective pairs of the vertices belonging to the first reference polyhedral network *Γ* by the vertices of the second reference polygonal network *B_v_*. The network *Γ* determines the general form of a building. The network *B_v_* defines the degree of the folding and discontinuity of a roof structure *Ω*. Both networks *Γ* and *B_v_* define the particular form and the general curvature of *Ω*.

The obtained results of the conducted research on the development of the rules governing the formation of the examined continuous roof shell structures and their modifications to the forms of discontinuous regular structures of many complete shells arranged in unconventional visually attractive patterns were implemented into the method’s algorithm. In particular, the relationships governing the position of each mesh *B_vij_* (*Ω_ij_*) in the network *B_v_* (structure *Ω*) and the values of the partition coefficients assigned to the vertices of each mesh were determined for the basic configuration CB and two derivative configurations CP1 and CP2. The position of each mesh *B_vij_* in *B_v_* defined by the appropriate formula related to the independent variables *i* and *j* was discussed. The complete tetrahedra *Γ_ij_*, closed spatial quadrangles *B_vij_* and ruled shell sectors *Ω_ij_* constituted the complex material used for creating the structures.

The invented formulas govern the mutual position of the vertices *A_ij_*, *B_ij_*, *C_ij_* and *D_ij_* of the subsequent meshes *B_vij_* (sectors *Ω_ij_*) in the network *B_v_* (structure *Ω*). The position results from: (a) the proportions assumed as functions of the positions of these vertices in relation to the respective points of the reference surface and the vertices of the *Γ* polyhedral network used, (b) the adopted formulas and integer values of the variables *i* and *j* related to the sequence of *B_vij_* in *B_v_*. The expected types of the geometric patterns formed by *B_vij_* (*Ω_ij_*) on the network *B_v_* (structure *Ω*) result from the relationships between the mutual positions of the vertices of the adjacent meshes *B_vij_*. The developed formulas lead to the respective mutual displacements of the adjacent meshes and their vertices along the side edges of the polyhedral structure *Γ*, which allows one to achieve the appropriate type and form of the final discontinuous roof structure.

## Data Availability

Data sharing not applicable.

## Nomenclature

[*x*,*y*,*z*]	global coordinate system
*Γ*	reference polyhedral network
*Γ_ij_*	single mesh of *Γ*
*W_ABij_*, *W_CDij_*, *W_ADij_* and *W_BCij_*	vertices of *Γ*
*u_ij_* and *v_ij_*	axes of *Γ*
*a_ij_*, *b_ij_*, *c_ij_* and *d_ij_*	side edges of *Γ*
*B_v_*	reference polygonal network
*B_vij_*	single mesh of *B_v_*
*A_ij_*, *B_ij_*, *C_ij_* and *D_ij_*	vertices of *B_vij_*
*ω*	reference regular surface
*S_Aij_*, *S_Bij_*, *S_Cij_* and *S_Dij_*	points of *ω*
*S_Aij_S_Bij_S_Cij_S_Dij_*	quadrangle with vertices at *A_ij_*, *B_ij_*, *C_ij_* and *D_ij_*
d*_SAij_*, d*_SBij_*, d*_SCij_* and d*_SDij_*	division coefficients of *W_ABij_*, *W_CDij_*, *W_ADij_*, *W_BCij_* by *S_Aij_*, *S_Bij_*, *S_Cij_*, *S_Dij_* and *A_ij_*, *B_ij_*, *C_ij_*, *D_ij_*
d*_Aij_*, d*_Bij_*, d*_Cij_* and d*_Dij_*	division coefficients of *W_ABij_, W_CDij_, W_ADij_*, *W_BCij_* by *A_ij_*, *B_ij_*, *C_ij_* and *D_ij_*
d*_S_*_\*Aij*_, d*_S_*_\*Bij*_, d*_S_*_\*Cij*_ and d*_S_*_\*Dij*_	double division coefficients of *W_ABij_*, *W_CDij_*, *W_ADij_*, *W_BCij_* by *S_Aij_*, *S_Bij_*, *S_Cij_*, *S_Dij_* and *A_ij_*, *B_ij_*, *C_ij_*, *D_ij_*
*Ω*	complex shell roof structure
*Ω_ij_*	complete shell unit of *Ω*
CB	basic continuous configuration of *Ω*
CPi	derivative discontinuous configuration of *Ω* derivative of CB
*i* and *j*	integer values expressing the position of *B_vij_* (*Ω _ij_*) in *B_v_* (*Ω*)
*k_C_*	integer constant
dd*_Aij_*, dd*_Bij_*, dd*_Cij_* and dd*_Dij_*	increments of the division coefficients d*_Aij_*, d*_Bij_*, d*_Cij_* and d*_Dij_*

## Appendix A

**Table A1:** The coordinates of the vertices *W_ABij_*, *W_CDij_*, *W_ADij_*, *W_BCij_* (for *i, j* = 1, 2, 3) of the polyhedral reference network *Γ*_1_.

Point	*x*-Coordinate [mm]	*y*-Coordinate [mm]	*z*-Coordinate [mm]
*W_AB_*_13_	3254.3	0.0	468.2
*W_CD_*_13_	5572.7	0.0	1487.4
*W_AD_*_13_	−435.4	−1054.6	12,007.1
*W_BC_*_13_	−435.4	1054.6	12,007.1
*W_AB_*_21_	−1100.0	−87.2	−996.2
*W_CD_*_21_	1100.0	−87.2	−996.2
*W_AD_*_21_	−4500.0	−4880.7	55,786.9
*W_BC_*_21_	0.0	2574.0	9677.1
*W_AB_*_31_	−1210.0	−353.3	−2063.5
*W_CD_*_31_	1210.0	353.3	−2063.5
*W_AD_*_31_	0.0	2574.0	9677.1
*W_BC_*_31_	0.0	4374.6	9074.6
*W_AB_*_23_	3589.7	−95.9	−580.8
*W_CD_*_23_	6173.6	−105.5	435.5
*W_AD_*_23_	−435.4	1054.6	12,007.1
*W_BC_*_23_	−480.0	3133.7	11,876.9
*W_AB_*_32_	1210.0	353.3	−2063.5
*W_CD_*_32_	3959.7	−389.5	−1713.3
*W_AD_*_32_	−110.0	2840.1	10,744.4
*W_BC_*_32_	−121.0	4847.4	10,188.4
*W_AB_*_33_	3959.7	−389.5	−1713.3
*W_CD_*_33_	6838.9	−429.4	−708.6
*W_AD_*_33_	−480.0	3133.7	11,876.9
*W_BC_*_33_	−529.1	5371.0	11,378.6

**Table A2:** The initial data defining the positions of the points *S_Aij_*, *S_Bij_*, *S_Cij_*, *S_Dij_* for *i*, *j* = 1, 2.

Ratio	Value
d*_SA_*_11_ = (*W_AB_*_11_,*W_AD_*_11_)\S*_A11_*	5.5000
d*_SB_*_11_ = (*W_AB_*_11_,*W_BC_*_11_)\S*_B11_*	5.5000
d*_SC_*_11_ = (*W_CD_*_11_,*W_AD_*_11_)\S*_C11_*	5.5000
d*_SD_*_11_ = (*W_CD_*_11_,*W_AD_*_11_)\S*_D11_*	5.5000
d*_SA_*_12_ = (*W_AB_*_12_,*W_AD_*_12_)\S*_A12_*	5.0000
d*_SB_*_12_ = (*W_AB_*_12_, *W_BC_*_12_)\S*_B12_*	5.0000
d*_SC_*_12_ = (*W_CD_*_12_, *W_BC_*_12_)\S*_C12_*	5.0000
d*_SD_*_12_ = (*W_CD_*_12_, *W_AD_*_12_)\S*_D12_*	5.0000
d*_SA_*_22_ = (*W_AB_*_22_,*W_AD_*_22_)\S*_A22_*	4.6667
d*_SB_*_22_ = (*W_AB_*_22_, *W_BC_*_22_)\S*_B22_*	4.6281
d*_SC_*_22_ = (*W_CD_*_22_, *W_AD_*_22_)\S*_C22_*	4.6667
d*_SD_*_22_ = (*W_CD_*_22_, *W_AD_*_22_)\S*_D22_*	4.6364

**Table A3:** The coordinates of the points defining the reference surface *ω*.

Point	*x*-Coordinate [mm]	*y*-Coordinate [mm]	*z*-Coordinate [mm]
*S_A11_*	4500.0	−4793.6	54,790.7
*S_B11_*	4500.0	4880.7	55,786.9
*S_C11_*	−4500.0	4793.6	54,790.7
*S_D11_*	−4500.0	−4880.7	55,786.9
*S_A12_*	−4500.0	−4880.7	55,786.9
*S_B12_*	−4500.0	4793.6	54,790.7
*S_C12_*	−13,517.1	4793.6	52,918.0
*S_D12_*	−13,517.1	−4793.6	52,918.0
*S_A13_*	−13,517.1	−4793.6	52,918.0
*S_B13_*	−13,517.1	4793.6	52,918.0
*S_C13_*	−21,737.1	4793.6	49,304.2
*S_D13_*	−21,737.1	−4793.6	49,304.2
*S_A21_*	4500.0	4880.7	55,786.9
*S_B21_*	4500.0	13,460.5	53,340.4
*S_C21_*	−4500.0	13,460.5	53,340.4
*S_D21_*	−4500.0	4793.6	54,790.7
*S_A31_*	4500.0	13,460.5	53,340.4
*S_B31_*	4500.0	21,957.5	50,497.3
*S_C31_*	−4500.0	21,957.5	50,497.3
*S_D31_*	−4500.0	13,460.5	53,340.4
*S_A22_*	−4500.0	4793.6	54,790.7
*S_B22_*	−4500.0	13,460.5	53,340.4
*S_C22_*	−13,675.6	13,605.3	52,270.2
*S_D22_*	−13,517.1	4793.6	52,918.0
*S_A23_*	−13,517.1	4793.6	52,918.0
*S_B23_*	−13,675.6	13,605.3	52,270.2
*S_C23_*	−22,104.0	13,660.9	49,061.5
*S_D23_*	−21,737.1	4793.6	49,304.2
*S_A32_*	−4500.0	13,460.5	53,340.4
*S_B32_*	−4500.0	21,957.5	50,497.3
*S_C32_*	−13,692.4	22,263.8	49,770.7
*S_D32_*	−13,675.6	13,605.3	52,270.2
*S_A33_*	−13,675.6	13,605.3	52,270.2
*S_B33_*	−13,692.4	22,263.8	49,770.7
*S_C33_*	−22,428.4	22,611.2	47,304.5
*S_D33_*	−22,104.0	13,660.9	49,061.5

**Table A4:** The division coefficients employed to calculate the coordinates of the *Bv* and *Ω* vertices for *i, j* = 1, 2.

Ratio	Value
d*_A11_* = (*W_AB11_*,*W_AD11_*)\*A_11_*	5.4000
d*_B11_* = (*W_AB11_*,*W_BC11_*)\*B_11_*	5.6000
d*_C11_* = (*W_CD11_*,*W_AD11_*)\*C_11_*	5.4000
d*_D11_* = (*W_CD11_*,*W_AD11_*)\*D_11_*	5.6000
d*_A12_* = (*W_AB12_*,*W_AD12_*)\*A_12_*	5.0910
d*_B12_* = (*W_AB12_*, *W_BC12_*)\*B_12_*	4.9090
d*_C12_* = (*W_CD12_*, *W_BC12_*)\*C_12_*	5.0910
d*_D12_* = (*W_CD12_*, *W_AD12_*)\*D_12_*	4.9090
d*_A22_* = (*W_AB22_*,*W_AD22_*)\*A_22_*	4.5834
d*_B22_* = (*W_AB22_*, *W_BC22_*)\*B_22_*	4.7114
d*_C22_* = (*W_CD22_*, *W_AD22_*)\*C_22_*	4.5834
d*_D22_* = (*W_CD22_*, *WAD_22_*)\*D_22_*	4.7190

**Table A5:** The values of the division coefficients (for *i, j* = 1, 2) used to estimate the folding of *Ω* (*B_v_*).

Ratio	Value
d*_S_*_11*A*11_ = (*W_AB_*_11_,*W_AD_*_11_)\(*S_A_*_11_,*A*_11_)	−0.1000
d*_S_*_11*B*11_ = (*W_AB_*_11_,*W_BC_*_11_)\(*S_B_*_11_,*B*_11_)	0.1000
d*_S_*_11*C*11_ = (*W_CD_*_11_,*W_BC_*_11_)\(*S_C_*_11_,*C*_11_)	−0.1000
d*_S_*_11*D*11_ = (*W_CD_*_11_,*W_AD_*_11_)\(*S_D_*_11_,*D*_11_)	0.1000
d*_S_*_12*A*12_ = (*W_AB_*_12_,*W_AD_*_12_)\(*S_A_*_12_,*A*_12_)	0.0910
d*_S_*_12*B*12_ = (*W_AB_*_12_, *W_BC_*_12_)\(*S_A_*_12_,*B*_12_)	−0.0910
d*_S_*_12*C*12_ = (*W_CD_*_12_, *W_BC_*_12_)\(*S_A_*_12_,*C*_12_)	0.0910
d*_S12D12_*= (*W_CD12_*, *W_AD12_*)\(*S_A12_*,*D_12_*)	−0.0910
d*_S22A22_* = (*W_AB22_*,*W_AD22_*)\(*S_A21_*,*A_22_*)	−0.0833
d*_S22B22_* = (*W_AB22_*, *W_BC22_*)\(*S_A21_*,*B_22_*)	0.0826
d*_S22C22_* = (*W_CD22_*, *W_BC22_*)\(*S_A22_*,*C_22_*)	−0.0833
d*_S22D22_* = (*W_CD22_*, *W_AD22_*)\(*S_A2_*_2_,*D_22_*)	0.0826

**Table A6:** The coordinates of the vertices *A_ij_*, *B_ij_*, *C_ij_*, *D_ij_* (for *i, j* = 1, 2, 3) of the basic configuration CB of the eaves edge net *B_v_*_1_.

Point	*x*-Coordinate [mm]	*y*-Coordinate [mm]	*z*-Coordinate [mm]
*A_11_*	4400.0	−4706.4	53,794.5
*B_11_*	4600.0	4880.7	55,786.9
*C_11_*	−4400.0	4706.4	53,794.5
*D_11_*	−4600.0	−4880.7	55,786.9
*A_12_*	*xD_11_*	*yD_11_*	*zD_11_*
*B_12_*	*xC_11_*	*yC_11_*	*zC_11_*
*C_12_*	−13,822.1	4880.7	53,871.7
*D_12_*	−13,212.2	−4706.4	51,964.4
*A_13_*	*xD_12_*	*yD_11_*	*zD_11_*
*B_13_*	*xC_12_*	*yC_11_*	*zC_11_*
*C_13_*	−21,240.6	4706.4	48,434.8
*D_13_*	−22,233.7	−4880.7	50,173.6
*A_21_*	*xB_11_*	*yB_11_*	*zB_11_*
*B_21_*	4400.0	13,218.5	52,370.0
*C_21_*	−4600.0	13,702.4	54,310.7
*D_21_*	*xC_11_*	*yC_11_*	*zC_11_*
*A_31_*	*xB_21_*	*yB_21_*	*zB_21_*
*B_31_*	4600.0	22,348.2	51,417.8
*C_31_*	−4400.0	21,566.7	49,576.8
*D_31_*	*xC_21_*	*yC_21_*	*zC_21_*
*A_22_*	*xD_21_*	*yD_21_*	*zD_21_*
*B_22_*	*xC_21_*	*yC_21_*	*zC_21_*
*C_22_*	−13,367.3	13,360.7	51,326.4
*D_22_*	*xC_12_*	*yC_12_*	*zC_12_*
*A_23_*	*xD_22_*	*yD_22_*	*zD_22_*
*B_23_*	*xC_22_*	*yC_22_*	*zC_22_*
*C_23_*	−22,608.0	13,906.3	49,928.3
*D_23_*	*xC_13_*	*yC_13_*	*zC_13_*
*A32*	*xD_31_*	*yD_31_*	*zD_31_*
*B_32_*	*xC_31_*	*yC_31_*	*zC_31_*
*C_32_*	−14,001.5	22,660.5	50,672.4
*D_32_*	*xC_22_*	*yC_22_*	*zC_22_*
*A_33_*	*xC_22_*	*yC_22_*	*zC_22_*
*B_33_*	*xC_32_*	*yC_32_*	*zC_32_*
*C_33_*	−21916.7	22,208.4	46,465.1
*D_33_*	*xC_23_*	*yC_23_*	*zC_23_*

**Table A7:** The values of the division coefficients (for *i, j* = 1, 2) used to precisely define the folding of *Ω* (*B_v_*).

Ratio	Value
d*_A_*_\*S*11_	−0.0182
d*_B_*_\*S*11_	0.0182
d*_C_*_\*S*11_	−0.0182
d*_C_*_\*S*11_	0.0182
d*_A_*_\*S*12_	0.0182
d*_B_*_\*S*12_	−0.0182
d*_C_*_\*S*12_	0.0182
d*_D\S12_*	−0.0182
d*_A\S22_*	−0.0178
d*_B\S22_*	0.0180
d*_C\S22_*	−0.0178
d*_D\S22_*	0.0178

**Table A8:** The coordinates of the vertices *A_ij_*, *B_ij_*, *C_ij_*, *D_ij_* (for *i, j* = 1, 2, 3) of the first modified configuration CP1 of the eaves edge net *B_v_*_1_.

Point	*x*-Coordinate [mm]	*y*-Coordinate [mm]	*z*-Coordinate [mm]
*A_11_*	4400.0	−4706.4	53,794.5
*B_11_*	4600.0	4880.7	55,786.9
*C_11_*	−4400.0	4706.4	53,794.5
*D_11_*	−4600.0	−4880.7	55,786.9
*A_12_*	−4400.0	−4706.4	53,694.9
*B_12_*	−4600.0	4880.7	55,786.9
*C_12_*	−13,212.2	4706.4	51,964.4
*D_12_*	−13,822.1	−4880.7	53,871.7
*A_13_*	−13,212.2	−4706.4	51,964.4
*B_13_*	−13,822.1	4880.7	53,871.7
*C_13_*	−21,240.6	4706.4	48,434.8
*D_13_*	−22,233.7	−4880.7	50,173.6
*A_21_*	4400.0	4706.4	53,794.5
*B_21_*	4600.0	13,702.4	54,310.7
*C_21_*	−4400.0	13,218.5	52,370.1
*D_21_*	*xB_12_*	*yB_12_*	*zB_12_*
*A_31_*	4400.0	13,218.5	52,370.1
*B_31_*	4600.0	22,348.2	51,417.8
*C_31_*	−4400.0	21,566.7	49,576.8
*D_31_*	−4600.0	13,702.4	54,310.7
*A_22_*	*xC_11_*	*yC_11_*	*zC_11_*
*B_22_*	*xD_31_*	*yD_31_*	*zD_31_*
*C_22_*	−13,367.3	13,360.7	51,326.4
*D_22_*	*xB_13_*	*yB_13_*	*zB_13_*
*A_23_*	*xC_12_*	*yC_12_*	*zC_12_*
*B_23_*	−13,983.9	13,850.0	53,213.9
*C_23_*	−21,599.9	13,415.6	48,194.7
*D_23_*	−22,233.7	4880.7	50,173.6
*A_32_*	−4400.0	13,218.5	52,370.1
*B_32_*	*xC_21_*	*yC_21_*	*zC_21_*
*C_32_*	−13,383.2	21,867.1	48,869.1
*D_32_*	*xB_23_*	*yB_23_*	*zB_23_*
*A_33_*	*xC_22_*	*yC_22_*	*zC_22_*
*B_33_*	−14,001.5	22,660.5	50,672.4
*C_33_*	−21,916.7	22,208.4	46,465.1
*D_33_*	−22,608.0	13,906.3	49,928.3

**Table A9:** The coordinates of the vertices *A_ij_*, *B_ij_*, *C_ij_*, *D_ij_* (for *i, j* = 1, 2, 3) of the second modified configuration CP2 of the eaves edge net *B_v_*_1_.

Point	*x*-Coordinate [mm]	*y*-Coordinate [mm]	*z*-Coordinate [mm]
*A_11_*	4400.0	−4706.4	53,794.5
*B_11_*	4600.0	4880.7	55,786.9
*C_11_*	−4400.0	4706.4	53,794.5
*D_11_*	−4600.0	−4880.7	55,786.9
*A_12_*	−4500.0	−4793.6	54,790.7
*B_12_*	−4300.0	4619.2	52,798.3
*C_12_*	−13,517.1	4793.6	52,918.0
*D_12_*	−12,907.3	−4619.2	51,010.0
*A_13_*	−12,602.3	−4532.1	50,057.1
*B_13_*	−13,212.2	4706.4	51,964.4
*C_13_*	−20,247.5	4532.1	46,696.0
*D_13_*	−21,240.6	−4706.4	48,434.8
*A_21_*	4500.0	4793.6	54,790.7
*B_21_*	4300.0	12,976.6	51,399.8
*C_21_*	−4500.0	13,460.5	53,340.4
*D_21_*	−4300.0	4619.2	52,798.3
*A_31_*	4200.0	12,734.7	50,429.5
*B_31_*	4400.0	21,566.7	49,576.8
*C_31_*	−4200.0	20,785.3	47,735.8
*D_31_*	−4400.0	13,218.5	52,370.1
*A_22_*	−4200.0	4532.1	51,802.1
*B_22_*	−4400.0	13,218.5	52,370.1
*C_22_*	−12,750.7	12,871.3	49,438.9
*D_22_*	−13,212.2	4706.4	51,964.4
*A_23_*	−12,907.3	4619.2	51,010.8
*B_23_*	−12,442.4	12,626.7	48,495.1
*C_23_*	−21,095.8	13,170.2	47,328.0
*D_23_*	−19,751.0	4444.9	45,826.6
*A_32_*	−4300.0	12,976.6	51,399.8
*B_32_*	−4100.0	20,394.5	46,815.3
*C_32_*	−13,074.1	21,470.3	47,967.4
*D_32_*	−12,442.4	12,626.7	48,495.1
*A_33_*	−12,134.1	12,382.0	47,551.3
*B_33_*	−12,764.9	21,073.6	47,065.8
*C_33_*	−19,870.0	20,597.1	43,107.6
*D_33_*	−20,591.8	12,924.8	46,461.2

## References

[1] Abramczyk J. (2017). Shell Free Forms of Buildings Roofed with Transformed Corrugated Sheeting.

[2] Reichhart A. (2002). Geometrical and Structural Shaping Building Shells Made Up of Transformed Flat Folded Sheets.

[3] Abramczyk J. (2020). Symmetric Free Form Building Structures Arranged Regularly on Smooth Surfaces with Polyhedral Nets. Symmetry.

[4] Abramczyk J. (2020). Innovative Building Forms Determined by Orthotropic Properties of Folded Sheets Transformed into Roof Shells. JASS.

[5] Abramczyk J. (2021). Folded Sheets as a Universal Material for Shaping Transformed Shell Roofs. Materials.

[6] Abramczyk J. Shape transformations of folded sheets providing shell free forms for roofing. Proceedings of the 11th Conference on Shell Structures Theory and Applications.

[7] Bathe K.J. (1996). Finite Element Procedures.

[8] Nilson V.E. (1962). Testing a light gauge steel hyperbolic paraboloid shell. Proc. ASCE J. Struct. Div..

[9] Winter G. (1974). Strength of thin steel compression flanges. Trans. ASCE.

[10] Petcu V., Gioncu D. Corrugated hypar structures. Proceedings of the I International Conference on Lightweight Structures in Civil Engineering.

[11] Parker J.E. (1969). Behavior of Light Gauge Steel Hyperbolic Paraboloid Shells. Ph.D. Thesis.

[12] Gergely P., Banavalkar P.V., Parker J.E. (1971). The analysis and behavior of thin-steel hyperbolic paraboloid shells. A Research Project Sponsored by the America Iron and Steel Institute, Report No. 338.

[13] Egger H., Fischer M., Resinger F. (1971). Hyperschale aus Profilblechen. Stahlbau.

[14] Davis J.M., Bryan E.R. (1982). Manual of Stressed Skin Diaphragm Design.

[15] Biswas M., Iffland J.S. Metal decks used to form hypar-shell panels. Proceedings of the 2nd Speciality Conference on Cold-Formed Steel Structures.

[16] Pottmann H., Asperi A., Kilian A., Hofer M. (2007). Architectural Geometry.

[17] Samyn P. Structures isobarres et isonoeuds. Proceedings of the 2nd International Conference on Space Structures.

[18] Reichhart A. Corrugated Deformed Steel Sheets as Material for Shells. Proceedings of the International Conference on Lightweight Structures in Civil Engineering.

[19] Reichhart A. Principles of designing shells of profiled steel sheets. Proceedings of the X International Conference on Lightweight Structures in Civil Engineering.

[20] Abramczyk J. (2019). Transformed Shell Roof Structures as the Main Determinant in Creative Shaping Building Free Forms Sensitive to Man-Made and Natural Environments. Buildings.

[21] Grey A. (1999). Modern Differential Geometry of Curves and Surfaces with Mathematica.

[22] Carmo M.P. (1976). Differential Geometry of Curves and Surfaces.

[23] Wei-Wen Y. (2000). Cold Formed Steel Design.

[24] Abbas I., Marin M., Saeed T. (2020). A GL model on thermo-elastic interaction in a poroelastic material using finite element method. Symmetry.

[25] Craciun E.M., Marin M., Pop N. (2020). Some Results in Green–Lindsay Thermoelasticity of Bodies with Dipolar Structure. Mathematics.

[26] Sharma A. (2015). Urban greenways: Operationalizing design syntax and integrating mathematics and science in design. Front. Arch. Res..

[27] Hasgül E. Space as configuration: Patterns of space and culture. Proceedings of the ARCHTHEO 2015_ 9th conference: Theory and History of Architecture.

[28] Eekhout M. Form as a Bridge between Architectural, Structural and Industrial Design. Proceedings of the 4th International colloqium on Structural Morphology IASS: Spatial Lattice and Tension Structures.

[29] Abramczyk J. (2021). Transformed Corrugated Shell Units Used as a Material Determining Unconventional Forms of Complex Building Structures. Materials.

